# Excision of an unusual tumour of oncocytic origin: a case report

**DOI:** 10.1093/jscr/rjab154

**Published:** 2021-05-04

**Authors:** William B Howden, Dakshika A Gunaratne, Richard M Gallagher

**Affiliations:** St Vincent's Hospital, Sydney, NSW, Australia

## Abstract

This case presents a 46-year-old woman presented with a 5-month history of a gradually enlarging, non-painful swelling her right medial canthus, with magnetic resonance imaging demonstrating a large tumour of her lacrimal sac. The lesion was subsequently excised and the histopathological diagnosis made of a rare primary oncocytoma of the lacrimal sac.

## CASE REPORT

**Figure 1:**
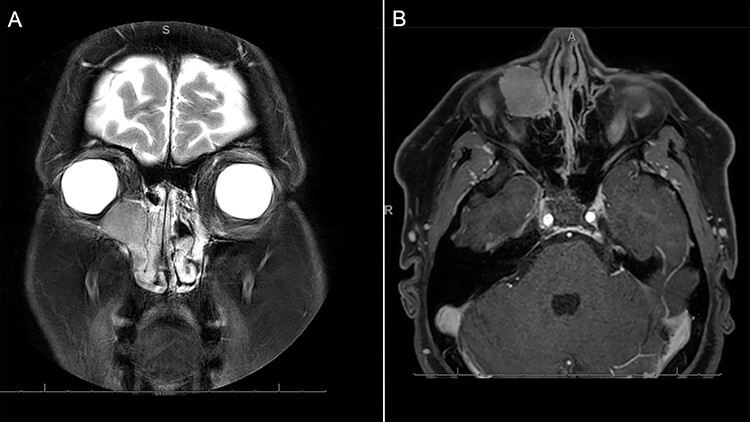
MRI demonstrating (**A**) coronal T2 demonstrating a homogenous, markedly hyperintense lesion of the right medial orbit, extending into the lacrimal duct. The contrast enhanced axial T1-weighted imaging demonstrates homogenous enhancement of the lesion with contrast.

**Figure 2:**
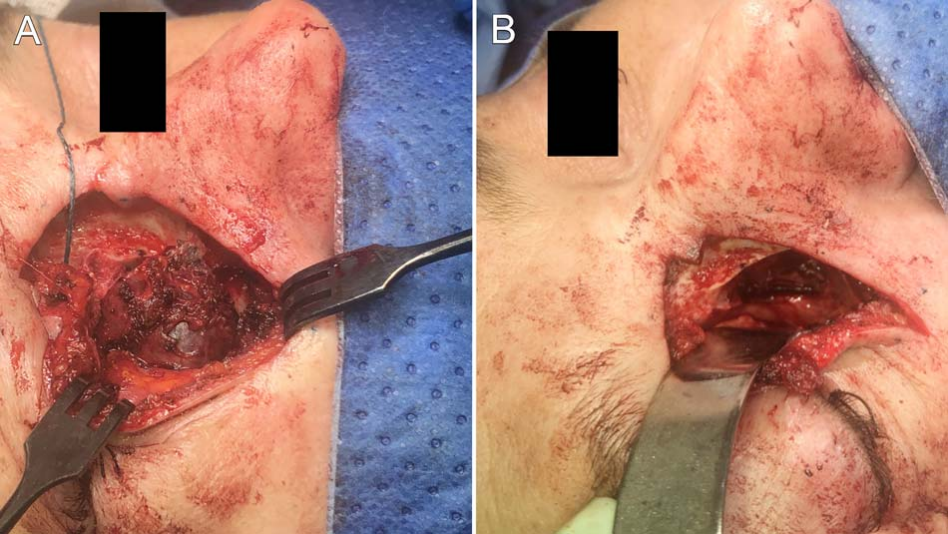
intraoperative images demonstrating (**A**) exposure of the lesion and (**B**) the post-excisional cavity.

A 46-year-old woman presented with a 5-month history of a gradually enlarging, non-painful swelling her right medial canthus. She reported mild right-sided nasal obstruction that predated the lesion but denied any history of epiphora, visual disturbance, epistaxis or facial pain/paraesthesia. On further questioning, she did report some discomfort with inferior gaze without diplopia. Her medical history was notable only for asthma. Examination revealed a palpable cystic lesion of the right medial canthus and infraorbital tissue extending over her right nasal bridge. There was no hyoglobus. Severe right septal deformity prevented visualization of the right inferior meatus, but the lateral nasal wall did not demonstrate deformity. Magnetic resonance imaging (MRI) demonstrated a 23 × 18.8 × 23.7 mm well-circumscribed cystic mass centred on the right lacrimal sac extending into the middle and inferior meatus. The lesion was isointense to muscle on T1 and markedly hyperintense on T2. It enhanced homogenously with contrast (Fig. 1). Expansile erosion of the right medial orbital wall, floor, anterior maxillary sinus and lateral nasal bone was noted with displacement of the medial rectus muscle inferolaterally (Fig. 2).

**Figure 3:**
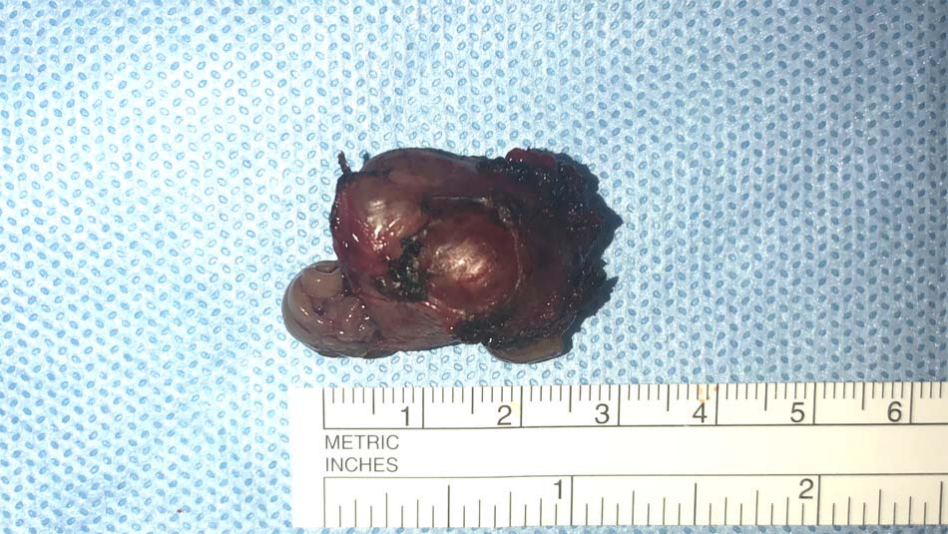
macroscopic image of the lesion demonstrating a 3 × 2 cm well-circumscribed, irregular soft tissue mass.

Excision of the lesion was undertaken via an extended right Lynch incision. Angular vessels were ligated and dissection proceeded in a subperiosteal plane superolaterally into the orbit and inferolaterally onto the anterior face of the maxilla. The anterior ethmoidal artery was ligated and the infraorbital nerve identified and preserved. The 3 × 2 cm mass was identified and carefully dissected free off surrounding bone and periorbital soft tissue (Fig. 3). The lesion encompassed the lacrimal sac and nasolacrimal duct and was followed into the inferior meatus. It was divided inferiorly at Hasner’s valve. A new dacrocystorhinostomy was created; Crawford nasolacrimal duct tubes introduced via the superior and inferior canaliculi and secured in the nasal cavity. Post-operative course was unremarkable, and the Crawford tubes were removed 6 weeks later.

Histopathology demonstrated a partially encapsulated, well-circumscribed tumour comprising large epithelial cells in closely packed clusters forming a larger nodule. The cells comprised abundant, granular eosinophilic cytoplasm, markedly variable nuclei with fine granular to pale chromatin and inconspicuous nucleoli—features consistent with an oncocytoma. The tumour extended into the nasolacrimal duct. There was no lymphovascular or perineural invasion. Immunohistochemistry was positive for cytokeratin-7 and a subpopulation of myoepithelial cells was highlighted with cytokeratin 5/6 and p63 at the periphery of the cell clusters. The oncocytic cells stained positive for SDHB.

## DISCUSSION

Primary tumours of the lacrimal sac are uncommon [1], most accounted for by squamous papilloma and carcinoma [2]. Oncocytes result from transformation of healthy epithelial cells into aged cells, which are swollen, degenerated and ‘functionally overtaxed’ [3]. They resemble an epithelial cell, which is larger in size with numerous mitochondria producing an abundant fine eosinophilic granular cytoplasm. Diagnosis relies on the detection of mitochondrial content with immunohistochemistry (SDHB or PTAH stains) or with the gold standard of electron microscopy [4]. Typically, oncocytic tumours of the head and neck are identified in the major salivary glands; however, they may arise from minor salivary gland subsites in the paranasal sinuses, nasopharynx and nasolacrimal duct. Very rarely do oncocytomas develop in the lacrimal sac [4]. Although oncocytomas are usually benign, they are often locally invasive and occasionally malignant in nature [2]. Benign oncocytomas are distinguished from their malignant counterparts (oncocytic carcinomas) by the presence of cellular pleomorphism with scattered mitosis; destructive, infiltrating growth; perineural or lymphovascular invasion and regional or distant metastasis. Due to the infrequency of oncocytic tumours of the lacrimal apparatus, their clinical behaviour is difficult to predict; however, they are more likely to be locally aggressive and have a higher incidence of malignancy than their major salivary gland counterparts [5]. Surgical resection via an open or endoscopic approach is the principal treatment modality. A recent review of sinonasal oncocytomas demonstrated recurrence in three of nine as oncocytic carcinomas following resection alone [4]. The role of adjuvant radiotherapy and chemotherapy is yet to be established.

## CONFLICT OF INTEREST STATEMENT

None declared.
